# Supplementary material for: Kinetics and Mechanisms of Saccharomyces boulardii Release from Optimized Whey Protein–Agavin–Alginate Beads under Simulated Gastrointestinal Conditions
Source: Bioengineering (Basel). 2022 Sep 9;9(9):460. doi: 10.3390/bioengineering9090460 (PMC9495568; doi:10.3390/bioengineering9090460)
Supplement: Supplementary file 1 [file bioengineering-09-00460-s001.zip › bioengineering-1859050-supplementary.pdf]

1. Supplementary material

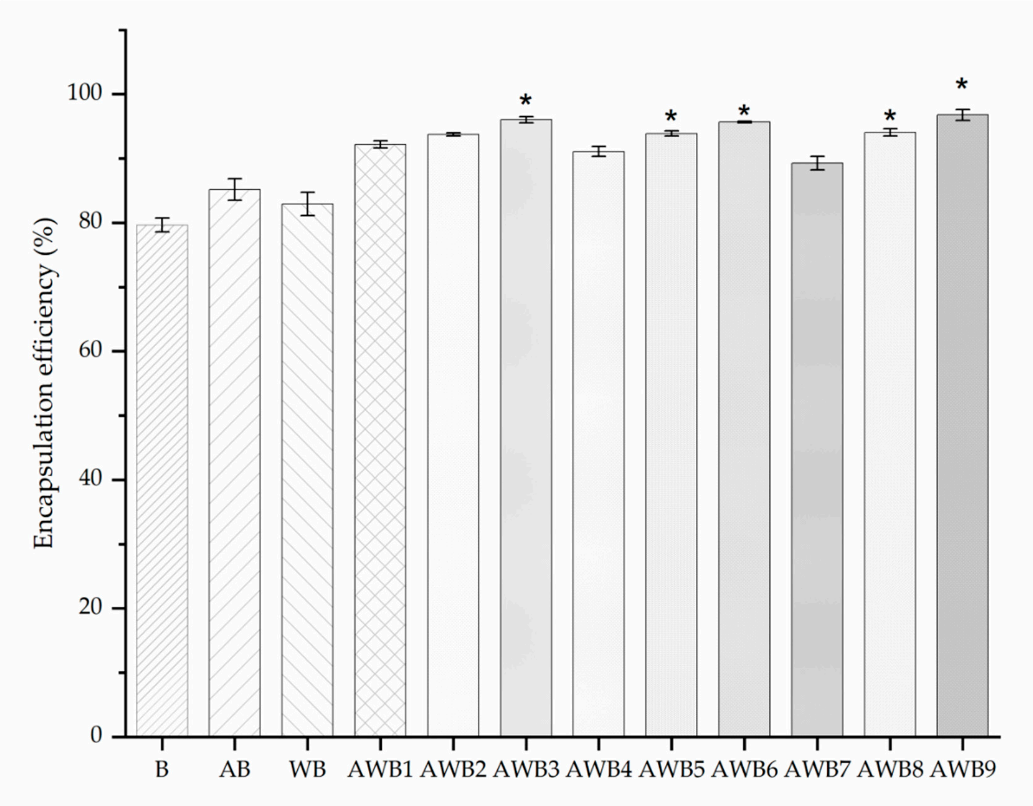

Figure S1. Encapsulation efficiency of *S. boulardii for* beads using agavins and whey protein as wall materials at different concentrations.

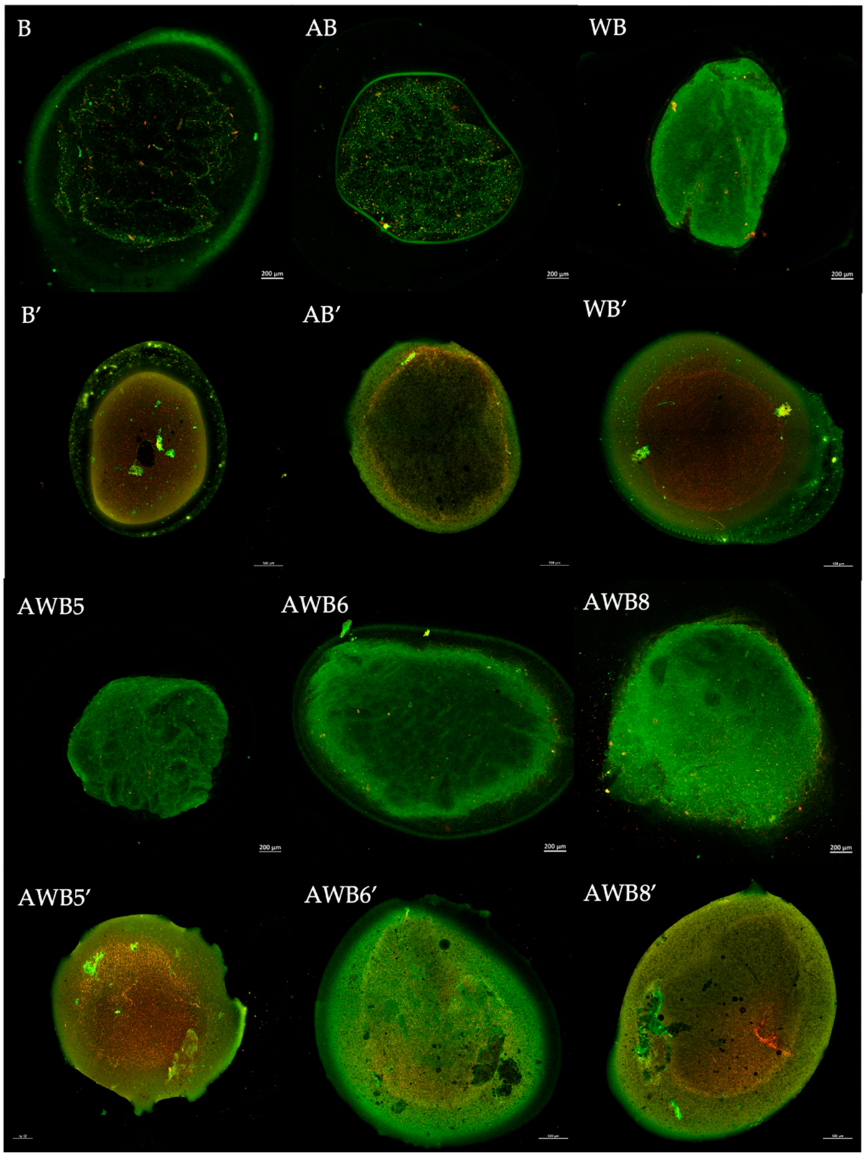

Figure S2. Confocal micrographs of optimized beads of *S. boulardii* under *in vitro* gastrointestinal digestion conditions for viability staining. Nomenclature without apostrophe indicates micrographs taken at minute 0 (salivary phase). Nomenclature with apostrophe indicates micrographs taken at minute 250 (intestinal phase).

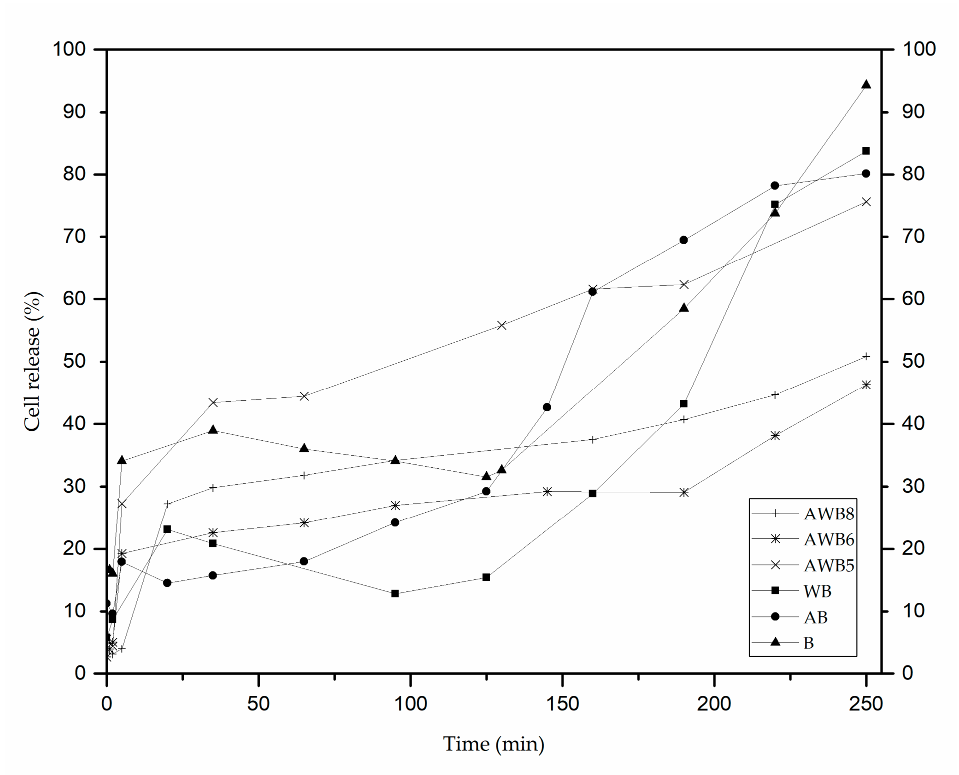

Figure S3. Percentage release of *S. boulardii* encapsulates during gastrointestinal digestion *in vitro*.

Table S1. Comparison of shape and dimensional parameters of *S. boulardii* encapsulates.

		Type of Bead							
		**B**	**AB**	**WB**	**AWB3**	**AWB5**	**AWB6**	**AWB8**	**AWB9**
**Area (mm^2)**	Mean	4.849_{d}	5.540_{a}	5.920_{c}	$5.405_{a,b}$	5.893_{c}	5.431_{a}	5.805_{c}	5.430_{a}
	SE	±0.04	±0.05	±0.07	±0.05	±0.05	±0.06	±0.06	±0.02
**Perimeter (mm)**	Mean	$9.439_{c,j}$	$9.396_{c,h}$	$9.516_{c,i}$	$9.168_{a,c,e,f,g}$	$9.441_{c,d,e}$	$9.082_{b,e,h,i,j}$	$9.152_{a,c,e,f,g}$	$8.931_{b,g}$
	SE	±0.14	±0.06	±0.09	±0.07	±0.06	±0.07	±0.06	±0.03
**Circularity**	Mean	0.692_{h}	$0.789_{b,e,g}$	$0.824_{a,f,g}$	$0.808_{a,e}$	$0.831_{a,c,e}$	$0.828_{a,c,e}$	0.871_{c}	$0.856_{c,d,f}$
	SE	±0.01	±0.00	±0.01	±0.01	±0.00	±0.01	±0.00	±0.00
**Solidity**	Mean	0.983_{a}	0.990_{b}	0.989_{b}	0.989_{b}	0.990_{b}	0.990_{b}	0.993_{b}	0.991_{b}
	SE	±0.00	±0.00	±0.00	±0.00	±0.00	±0.00	±0.00	±0.00

SE: Standard error of the mean. Note: values from the same row and subtable that do not share the same subscript are significantly different in the multiple comparison test Tukey, $p<0.05$.

Table S2. Comparison of internal structure parameters of *S. boulardii* encapsulates.

		Type of Bead					
		**B**	**AB**	**WB**	**AWB5**	**AWB6**	**AWB8**
**Second angular momentum**	Mean	0.964b,c	0.944a	0.963b	0.945a	0.950a,b	0.956a,b
	SE	±0.00	±0.00	±0.00	±0.00	±0.01	±0.00
**Contrast**	Mean	1008.881b,c	1584.216a	1051.047b	1541.675a	1424.428a,b	1239.540a,b
	SE	±53.69	±55.19	±20.13	±194.70	±132.27	±49.68
**Correlation**	Mean	0.000370b,c	0.000232a	0.000354b	0.000253a	0.000264a	0.000301a,b
	SE	±0.00	±0.00	±0.00	±0.00	±0.00	±0.00
**Entropy**	Mean	0.109b,c	0.158a	0.113b	0.155a	0.144a,b	0.130a,b
	SE	±0.00	±0.01	±0.00	±0.02	±0.01	±0.01
**Fractal dimension**	Mean	1.561c	1.638a	1.574b,c	1.627a	1.615a,b	1.602a,b,c
	SE	±0.01	±0.01	±0.00	±0.02	±0.01	±0.01
**Lacunarity**	Mean	0.039a,b	0.028b	0.031a,b	0.043a	0.034a,b	0.032a,b
	SE	±0.00	±0.00	±0.00	±0.01	±0.00	±0.00

SE: Standard error of the mean. Note: values from the same row and subtable that do not share the same subscript are significantly different in the multiple comparison test Tukey, $p<0.05$.

Table S3. Comparison of internal structure parameters of *S. boulardii* encapsulates.

		Types of Beads					
		**B**	**AB**	**WB**	**AWB5**	**AWB6**	**AWB8**
**Second angular momentum**	Mean	0.0003_{b}	0.0002_{b}	0.0003_{b}	0.0008_{a}	0.0007_{a}	0.0004_{b}
	SE	±0.000	±0.000	±0.000	±0.001	±0.000	±0.000
**Contrast**	Mean	420.942_{a}	426.818_{a}	315.955_{a}	332.509_{a}	169.706_{b}	324.542_{a}
	SE	±29.102	±31.257	±29.355	±38.740	±11.348	±24.209
**Correlation**	Mean	$0.000970_{c,e}$	0.000862_{c}	$0.001055_{c,d}$	0.001713_{a}	0.002480_{b}	$0.001259_{a,c}$
	SE	±0.000	±0.000	±0.000	±0.000	±0.000	±0.000
**Inverse differential momentum**	Mean	0.097_{b}	0.080_{b}	0.096_{b}	0.148_{a}	0.143_{a}	0.094_{b}
	SE	±0.005	±0.003	±0.005	±0.011	±0.004	±0.003
**Entropy**	Mean	$8.407_{b,c}$	8.590_{c}	$8.410_{b,c}$	7.588_{a}	7.534_{a}	8.212_{b}
	SE	±0.088	±0.050	±0.074	±0.117	±0.060	±0.064
**SDBC**	Mean	2.345_{a}	2.411_{b}	2.357_{a}	2.359_{a}	2.348_{a}	2.398_{b}
	SE	±0.005	±0.006	±0.006	±0.009	±0.005	±0.005

SE: Standard error of the mean. Note: values from the same row and subtable that do not share the same subscript are significantly different in the multiple comparison test Tukey, $p<0.05$.
